# OTUB1 regulates ferroptosis to inhibit myoblast differentiation into myotubes by deubiquitinating P62

**DOI:** 10.1038/s41598-024-66868-3

**Published:** 2024-07-08

**Authors:** Limin Wei, Yanhong Li, Helin Tan, Yue Peng, Qian Liu, Tingting Zheng, Feng Li, Zhongxian Xu

**Affiliations:** 1 Chongqing Key Laboratory of High Active Traditional Chinese Drug Delivery System, Chongqing Medical and Pharmaceutical College, Chongqing, 401331 China; 2 Key Laboratory of Endemic and Ethnic Diseases, Ministry of Education & Key Laboratory of Medical Molecular Biology of Guizhou Province, & Collaborative Innovation Center for Prevention and Control of Endemic and Ethnic Regional Diseases Co-Constructed By the Province and Ministry, Guizhou Medical University, Guiyang, 550004 China; 3 Key Laboratory of Southwest China Wildlife Resources Conservation (Ministry of Education), China West Normal University, Nanchong, 637009 China

**Keywords:** OTUB1, Ferroptosis, Autophagy, Skeletal muscle, Myogenesis, Cell biology, Autophagy

## Abstract

As the largest organ in the human body, skeletal muscle is essential for breathing support, movement initiation, and maintenance homeostasis. It has been shown that programmed cell death (PCD), which includes autophagy, apoptosis, and necrosis, is essential for the development of skeletal muscle. A novel form of PCD called ferroptosis is still poorly understood in relation to skeletal muscle. In this study, we observed that the activation of ferroptosis significantly impeded the differentiation of C2C12 myoblasts into myotubes and concurrently suppressed the expression of OTUB1, a crucial deubiquitinating enzyme. OTUB1-silenced C2C12 mouse myoblasts were used to investigate the function of OTUB1 in ferroptosis. The results show that OTUB1 knockdown in vitro significantly increased C2C12 ferroptosis and inhibited myogenesis. Interestingly, the induction of ferroptosis resulting from OTUB1 knockdown was concomitant with the activation of autophagy. Furthermore, OTUB1 interacted with the P62 protein and stabilized its expression by deubiquitinating it, thereby inhibiting autophagy-dependent ferroptosis and promoting myogenesis. All of these findings demonstrate the critical role that OTUB1 plays in controlling ferroptosis, and we suggest that focusing on the OTUB1-P62 axis may be a useful tactic in the treatment and prevention of disorders involving the skeletal muscle.

## Introduction

Programmed cell death (PCD), such as apoptosis, autophagy, and the recently discovered programmed necrosis (also known as necroptosis), plays a pivotal role in eliminating dead, dispensable, or senescent cells during organismal and organ development/regeneration^1,2^. Skeletal muscle accounts 40% of the total animal body weight and serves as a vital organ in the human body^3^. It not only supports movement but also plays an important role in regulating systemic metabolism^4^. Several studies have reported that muscle cell death manifests through various mechanisms, including apoptosis^5^, necrosis^6^, and autophagy^7^. The development of skeletal muscle is precisely regulated by PCD, and disruption of these pathways results in muscle atrophy, weakness, and disease^8^. Skeletal muscle necrosis can be observed in diverse pathological states, encompassing muscular dystrophy and ischemia^6^. However, acute or physiological injuries can trigger skeletal muscle apoptosis through several pivotal molecules, including the anti-apoptotic oncoproteins Bcl2 and Mcl-1 and the death receptors CD95 (FAS) and CD178 (FASL)^9,10^. In addition, autophagy is crucial for preserving the homeostasis of skeletal muscle. High levels of autophagy lead to excessive protein degradation, resulting in skeletal muscle atrophy; however, insufficient autophagy results in massive protein accumulation, which can lead to muscle toxicity^11^. These findings imply that PCD is crucial for preserving the bulk and homeostasis of skeletal muscle. However, the role of ferroptosis, a recently discovered form of PCD, in skeletal muscle development remains poorly understood.

The newly identified regulated cell death process known as ferroptosis is typified by lipid peroxidation that is dependent on iron; this pathway exhibits distinct morphological, biochemical, and genetic features compared to apoptosis, necroptosis and autophagy^12,13^. Ferroptosis is closely linked to the pathophysiological mechanisms of numerous illnesses, such as cardiomyopathy caused by ischemia/reperfusion^14^, cancer^15^, neurodegenerative diseases^16^, and inflammation^17^. Further investigation of the underlying mechanisms of ferroptosis could result in novel avenues for disease diagnosis and treatment.

Recent research has consistently shown that skeletal muscle diseases and ferroptosis are significantly correlated^18^. In muscle specimens obtained from elderly individuals with sarcopenia, relevant changes were observed in ferroptosis-associated genes, including heme oxygenase-1 (HO-1), spermidine/spermine N1-acetyltransferase 1, and prostaglandin-endoperoxide synthase^19^. Furthermore, transferrin receptor 1 is a critical regulator of the activation, proliferation, and maintenance of satellite cells, and its expression is markedly downregulated in aged skeletal muscle^20^. Concurrently, it was found that p53/SLC7A11 axis activation can cause ferroptosis in C2C12 myoblasts and prevent them from differentiating into myotubes, which can accelerate the progression of sarcopenia^21^. However, this process can be efficiently reversed by treatment with DFO plus the ferroptosis inhibitor ferrostatin-1. Ferroptosis may thus be crucial in controlling diseases of the skeletal muscle. Revealing novel ferroptosis factors and elucidating their effects on skeletal muscle development and physiology could have profound implications for advancing human health.

Otubain1 (OTUB1) is a significant deubiquitinase belonging to the OTU domain family^22^. Previous comprehensive studies have shown the pivotal role of OTUB1 in cancer initiation, DNA damage response, energy metabolism, and pulmonary fibrosis^23–25^. Furthermore, multiple studies have demonstrated that OTUB1 has a potential targeted regulatory association with several ferroptosis-related regulatory factors. Liu, et al. demonstrated the crucial role of OTUB1 in modulating the stability of SLC7A11, a pivotal regulator of ferroptosis, and its effects on human tumor cell proliferation^26^. Yan, et al. discovered that OTUB1 encourages the growth and migration of gastric cancer cells via inhibiting the Hippo pathway through deubiquitination and stabilizing the protein expression of YAP^24^. The role of YAP as a negative regulator of ferroptosis is widely acknowledged, and endogenous glutamate effectively suppresses ferroptosis signaling by modulating ADCy10-dependent YAP activity^27^. These studies collectively indicate the potential regulatory association between OTUB1 and ferroptosis.

In this study, we examined OTUB1's function in controlling the muscle cell ferroptosis process. Specifically, our findings demonstrate that OTUB1 controls ferroptosis in C2C12 myoblasts via interacting with P62; OTUB1 thus plays a pivotal role in muscle differentiation and maintenance. The findings of our study may offer valuable insight into the regulatory role of OTUB1 in the pathogenesis and clinical prevention of skeletal muscle diseases.

## Materials and methods

### Cell culture

The mouse myoblast cell line C2C12 was obtained from Procell Biotechnology Co., Ltd. (Wuhan, China) and cultivated in DMEM (Gibco, Grand Island, NY, USA) supplemented with 10% fetal bovine serum (FBS, Gibco) and 0.1% antibiotic–antimycotic (Solarbio, Beijing, China). The cells were kept in a humidified environment with 95% air and 5% CO_2_ at 37 °C. After C2C12 myoblasts reached 80% confluency, they were cultured in full medium and then switched to DMEM supplemented with 2% horse serum (Gibco) to accomplish myotube differentiation.

### RNA expression analysis

RNAiso plus reagents were used to extract total RNA from C2C12 cells in accordance with the manufacturer's instructions (Takara). A Nanodrop 2000 nucleic acid and protein detector (Thermo Fisher, Boston, Massachusetts, USA) was then used to evaluate the overall RNA quality. After that, high-quality RNA samples were reverse transcribed into complementary DNA (cDNA) using a Takara gDNA Eraser Kit and the PrimeScript RT reagent. TB Green PCR Master Mix (Takara) was used in quantitative real-time PCR (qRT-PCR) for the gene expression analysis of the cDNA samples in accordance with the suggested methodology from the manufacturer. Table 1 lists the sequences of the amplification primers that were created with Primer 5 software. The 2^−ΔΔCt^ method was employed to examine the qRT-PCR data, with the housekeeping gene tubulin serving as the internal reference.

**Table 1:** qRT-PCR primers used in this study.

Genes	Sequences (5’–3’)	Genebank number
OTUB1	F: GCTGTGCAGAATCCTCTGGT	NM_134150.2
	R: AAGCCAAACGCTCGGTAGAA	
MyoG	F: ACTCCCTTACGTCCATCGTG	NM_031189.2
	R: CAGGACAGCCCCACTTAAAA	
MyoD	F: AGCACTACAGTGGCGACTCA	NM_010866.2
	R: GGCCGCTGTAATCCATCA	
FTH1	F: CAAGTGCGCCAGAACTACCA	NM_010239.2
	R: CAAGTGCGCCAGAACTACCA	
GPX4	F: CCAAAGTCCTAGGAAACGCCC	NM_001037741.4
	R: CCCGGGTTGAAAGGTTCAGG	
SLC7A11	F: CGTCATCGGATCAGGCATCT	XM_006500777.4
	R: TCCAGGGCAAAAAGTGACAG	
Tubulin	F: CTGGGCTCGCCTAGATCAC	NM_205518.2
	R:AGACCACAACTTTCTGTGTTGGA	

### Western blot analysis

Total proteins were collected from different treatment groups using protein lysis buffer, and the concentration was measured using a bicinchoninic acid (BCA) protein assay kit (BestBio, Shanghai, China). Total proteins were separated by 10% SDS-PAGE and then transferred to a 0.2 μm polyvinylidene fluoride (PVDF) membrane (Millipore Corporation, Billerica, MA, USA). The membranes were blocked with Quickblock closed solution (Beyotime, Shanghai, China) and incubated with primary antibodies specific. The next day, anti-rabbit or anti-mouse horseradish peroxidase conjugated secondary antibodies also were used to incubate the PVDF membranes. Finally, protein bands were enhanced by the chemiluminescence (ECL) system (Beyotime, Shanghai, China) and the protein expression contents were calculated by the ImageJ software (National Health Institute, Bethesda, MD, USA).

The primary antibodies used in this experiment were as follows: OTUB1 (Santa Cruz Biotechnology, Inc., Heidelberg, Germany); MyHC (Santa Cruz Biotechnology); LC3B (Sigma, St. Louis, MO, USA); MyoG (Biorbyt, Cambridge, UK); MyoD (Abcam, Cambridge, UK); Beclin-1 (Cell Signaling Technology, Massachusetts, USA); and tubulin (Zen Bioscience, Chengdu, China). The second antibodies used in this experiment were as follows: goat anti-rabbit HRP (Zen Bioscience); goat anti-mouse HRP (Zen Bioscience), and TRITC-goat anti-rabbit IgG (Zen Bioscience).

### Examination of cell viability and death

Using microscopic examination and propidium iodide (PI) staining, C2C12 cell death was examined. The Cell Counting Kit-8 (CCK-8) assays (Sigma-Aldrich, St. Louis, MO, USA) were used to assess the viability of the cells. 96-well plates were seeded with C2C12 cells in accordance with usual procedures. Following a designated protocol, 10 μl of CCK-8 reagent was introduced into every well, and the cells were cultured for two hours at 37 °C with 5% CO_2_. Using a spectrophotometer designed specifically for measuring microplate absorbance, the absorbance was determined at 450 nm (Thermo Fisher Scientific, Waltham, Massachusetts, USA).

### Determination of lipid peroxidation, GSH, and *iron* content

Lipid peroxidation detection kits (MAK085 and G4501, Sigma) were utilized in accordance with the manufacturer's instructions to measure the amounts of lipid peroxidation products, MDA and GSSG, in cell lysates. A GSH assay kit was used to determine the relative quantity of GSH in cell lysates (CS0260, Sigma). An iron assay kit was used to measure the relative iron content of cell lysates (G4501, Sigma).

### Double-labeled adenovirus mRFP-GFP-LC3 transfection

In accordance with the manufacturer's instructions, C2C12 cells were grown on a confocal plate for two days before being transfected with the mRFP-GFP-LC3 adenovirus (Hanbio, China). The cells were treated, then fixed in 4% paraformaldehyde, rinsed with phosphate-buffered saline (PBS), and analyzed using a Zeiss confocal microscope (Oberkochen, Germany).

### Production of transfectants with OTUB1 silenced and overexpressed

A pcDNA3.1 expression vector expressing mouse OTUB1-Flag (OriGene, Rockville, MD, USA) for overexpression and mouse OTUB1 shRNA (Sigma-Aldrich) for silencing was used to transfect C2C12 cells in a stable manner. Following the manufacturer's instructions, stable clones were produced using the Lipofectamine 3000 transfection reagent (Invitrogen, Carlsbad, USA).

### Immunofluorescence and confocal microscopy

After 48 h of transfection, C2C12 cells were grown in 24-well plates and fixed for 15 min in 4% formaldehyde. After three PBS washes, the cells were permeabilized for ten minutes using 0.5% Triton X-100 (Sigma). The cells were then blocked for one hour with goat serum (Gibco) and treated with the primary antibody in PBS‐1% goat serum (Gibco) for the entire night at 4 °C. After that, the cells were treated for 60 min without light with a fluorescent secondary antibody. DAPI staining (Sigma) was used to see the cell nuclei. To visualize cells, a fluorescent microscope (Olympus, Melville, NY, USA) was used.

### Statistical analysis

The independent-sample t-test was utilized to determine statistical significance between the experimental and control groups, and Duncan's technique was applied for multiple comparisons. All data are presented as the mean ± standard error of the mean (SEM) for a minimum of three independent repeats. The software SPSS v20 (IBM Corp, Armonk, NY, USA) was used for statistical analyses, with a significance level of *p* < 0.05.

## Results

### Role of ferroptosis in skeletal muscle differentiation

**Figure 1:**
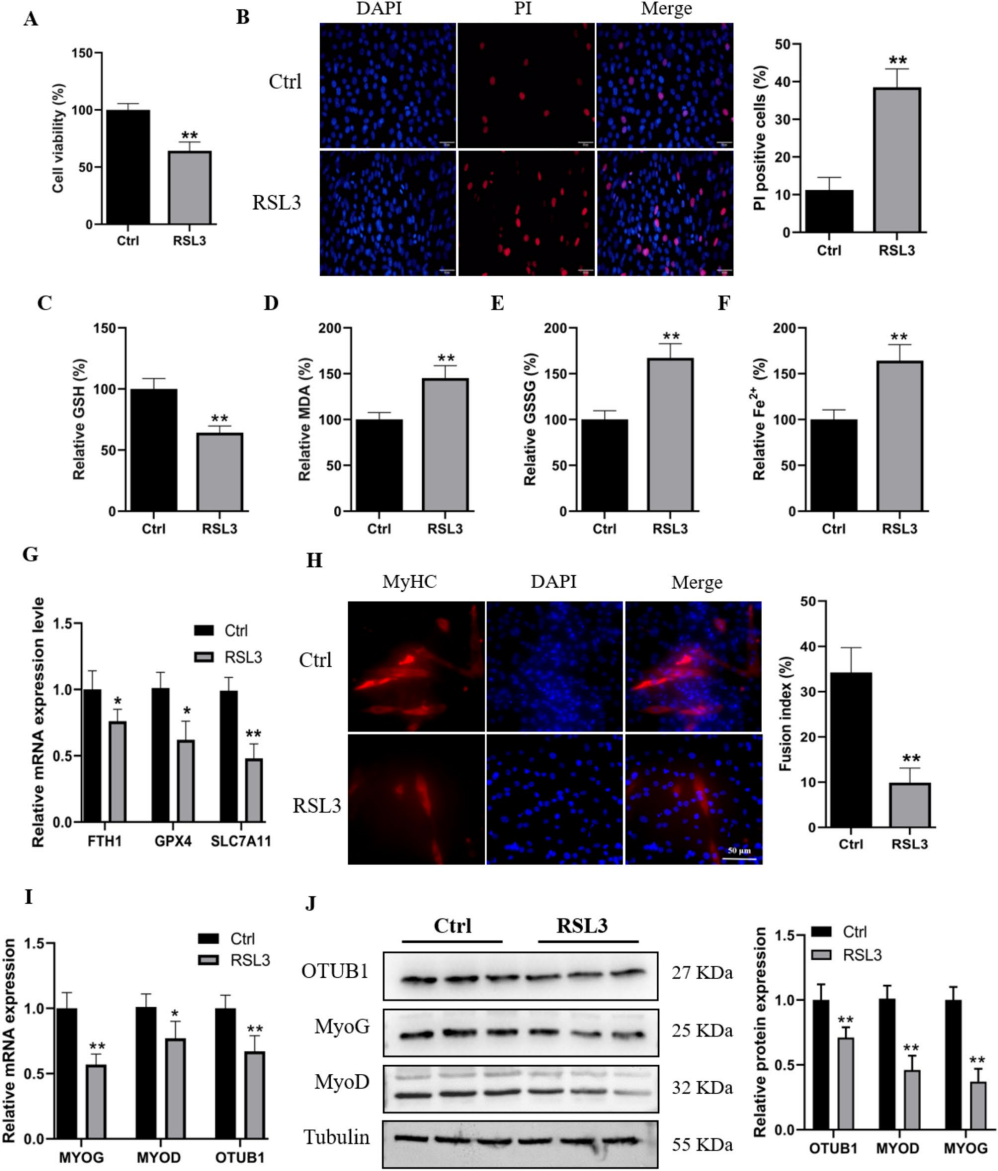
Ferroptosis inhibits the differentiation of C2C12 myoblasts into myotubes and downregulates the expression of OTUB1. (**A**) Using CCK-8 tests, cell viability was evaluated after a 24-h treatment with DMSO or RSL3. (**B**) PI staining was used to detect cell survival following 24-h treatment with either RSL3 or DMSO. (**C**–**F**) The levels of GSH, MDA, GSSG, and Fe^2+^ were assessed after treatment with RSL3 or DMSO for 24 h. (**G**) Relative mRNA expression levels of FTH1, GPX4 and SLC7A11 were assessed after treatment with RSL3 or DMSO for 24 h. (**H**) After 24 h of treatment with RSL3 or DMSO, C2C12 cells were stained with DAPI (nuclei) and MyHC antibodies. The bar graph shows the myotube fusion index. (**I**,**J**) The cells were induced to differentiate for 24 h, the MyoD, MyoG, and OTUB1 mRNA and protein levels following a 24 h after treatment with RSL3 or DMSO were examined using qRT-PCR and western blot. The data represent the means ± SEM (n = 3 independent cell cultures). * *p* < 0.05; ** *p* < 0.01.

To investigate the effects of ferroptosis on myogenesis, C2C12 cells were cultured in differentiation medium for 24 h, followed by treatment with either ferroptosis inducer RSL3 or DMSO (control) for an additional 24 h. CCK-8 assay results revealed that compared to control conditions, RSL3 treatment significantly reduced C2C12 cell viability (Fig. 1A). PI staining also revealed a significant increase in C2C12 myoblast cell death following RSL3 treatment (Fig. 1B). Crucial steps in the process of ferroptosis include Fe^2+^ buildup, lipid peroxidation, and GSH depletion. As anticipated, the induction of ferroptosis events was observed upon treatment with RSL3 (Fig. 1C–F). qPCR analysis indicated that RSL3-treated C2C12 myoblasts had significantly decreased mRNA expression levels of FTH1, GPX4 and SLC7A11 (Fig. 1G).

In order to get additional insight into the function of ferroptosis in myogenesis, we looked at how RSL3 treatment affected myoblast differentiation into myotubes. MyHC immunofluorescence assay results revealed a significant reduction in C2C12 myoblast differentiation into myotubes following RSL3 treatment compared to control conditions (Fig. 1H). Additionally, treatment with the ferroptosis inducer RSL3 markedly downregulated the expression of the myogenic genes MyoG and MyoD and decreased OTUB1 expression levels (Fig. 1I,J). These findings suggest a close association between ferroptosis and myoblast differentiation, and the regulation of the ferroptosis pathway by OTUB1 may play a crucial role in skeletal muscle development.

### OTUB1 regulates ferroptosis in skeletal muscle

**Figure 2:**
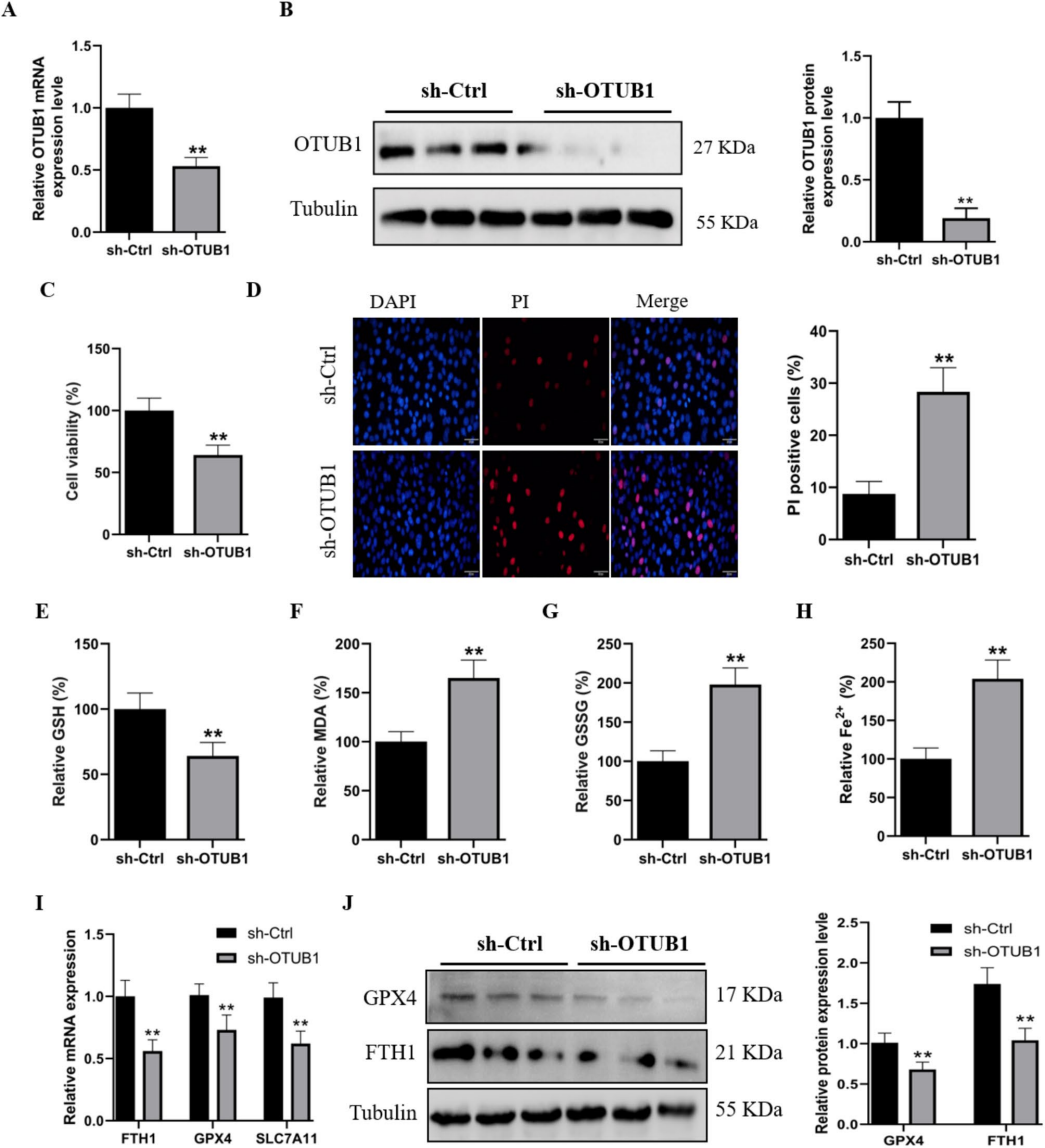
OTUB1 silencing activates ferroptosis in C2C12 cells. (**A**) qRT-PCR analysis of OTUB1 mRNA expression levels in OTUB1-silenced and shCtrl cells. (**B**) western blot analysis of OTUB1 protein levels in OTUB1-silenced and shCtrl cells. (**C**) Cell viability was detected by CCK-8 assays in OTUB1-silenced and shCtrl cells. (**D**) PI staining was used to detect cell survival in OTUB1-silenced and shCtrl cells. (**E**–**H**) The levels of GSH, MDA, GSSG, and Fe^2+^ were determined in OTUB1-silenced and shCtrl cells, sh-OTUB1 group as control. (**I**) qRT-PCR was used to detect the mRNA expression levels of FTH1, GPX4, and SLC7A11 in OTUB1-silenced and shCtrl cells. (**J**) The protein expression levels of FTH1 and GPX4 in OTUB1-silenced and shCtrl cells were measured by western blotting. The data represent the means ± SEM (n = 3 independent cell cultures). * *p* < 0.05; ** *p* < 0.01.

C2C12 cells were transfected with OTUB1 shRNA to inhibit OTUB1 expression in order to study the impact of OTUB1 on ferroptosis. Compared to control shRNA transfection, transfection with shRNA targeting OTUB1 mRNA resulted in a 55% reduction in endogenous OTUB1 mRNA expression. (Fig. 2A,B). The CCK-8 assay results showed that silencing OTUB1 significantly decreased C2C12 cell viability (Fig. 2C). PI staining also indicated increased cell death in OTUB1-silenced cells (Fig. 2D). A decrease in GSH levels, along with increases in MDA, GSSG, and Fe^2+^ content, suggests that silencing OTUB1 induces ferroptosis events (Fig. 2E–H). We also examined the expression levels of the ferroptosis marker genes FTH1, GPX4 and SLC7A11. Our results showed that compared to C2C12 cells transfected with control shRNA, those transfected with OTUB1 shRNA exhibited significantly lower mRNA expression levels of FTH1, GPX4 and SLC7A11 (Fig. 2I). The protein expression levels of FTH1 and GPX4 were similarly shown to be considerably lower in OTUB1-silenced cells, according to Western blot data (Fig. 2J). These findings potentially suggest that OTUB1 regulates ferroptosis in C2C12 myoblasts.

### OTUB1 regulates myogenesis through the ferroptosis pathway

**Figure 3:**
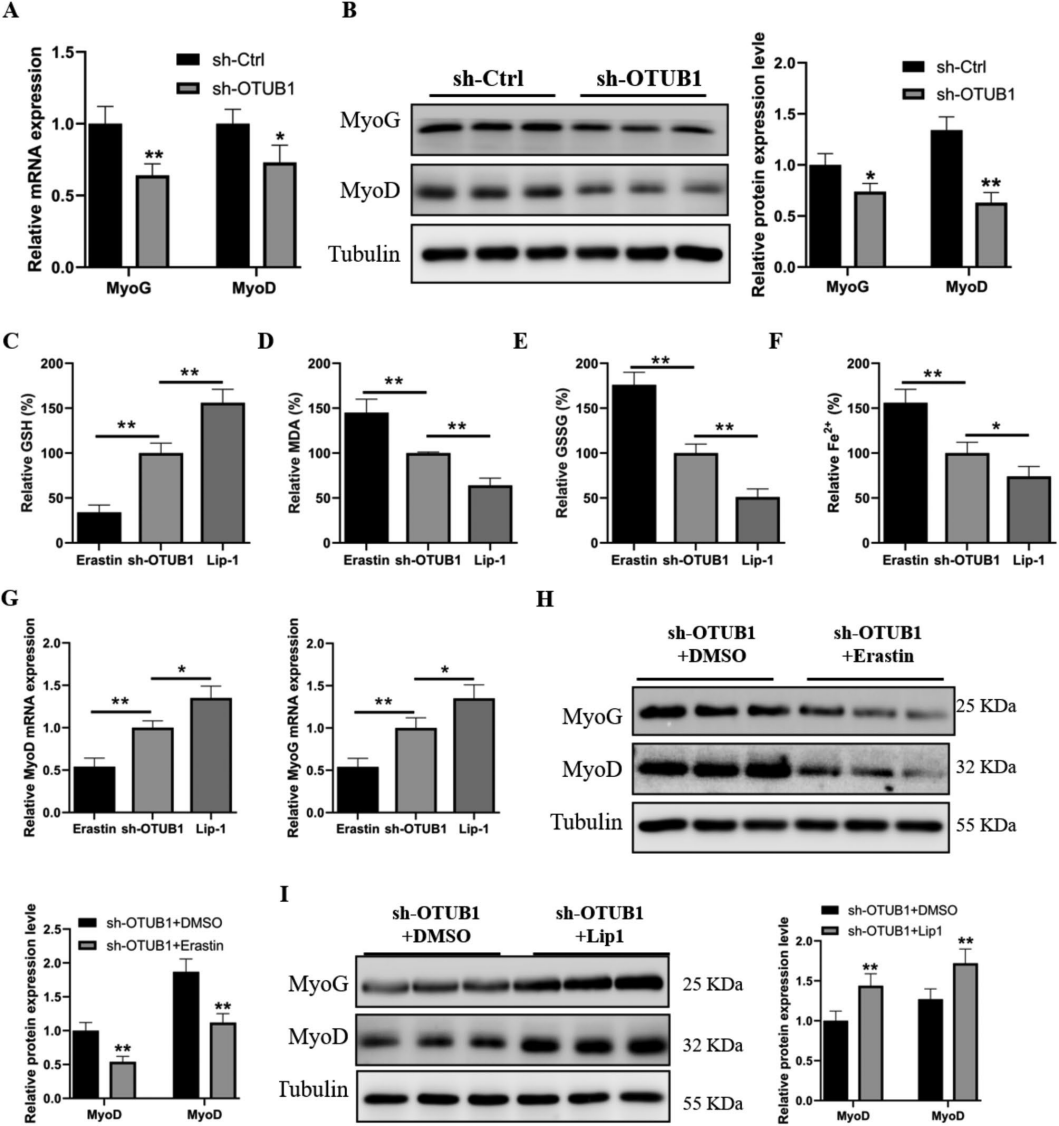
OTUB1 mediates ferroptosis to regulate skeletal muscle development. (**A**,**B**) MyoG and MyoD mRNA and protein expression levels were measured in OTUB1-silenced and shCtrl cells using Western blotting and qRT-PCR. (**C**–**F**) The levels of GSH, MDA, GSSG, and Fe^2+^ were determined after treating OTUB1-silenced cells with erastin or Lip-1. (**G**) qRT-PCR was used to detect the mRNA expression levels of MyoG and MyoD after treating OTUB1-silenced cells with erastin or Lip-1. (**H**) Western blotting was used to detect the protein expression levels of MyoG and MyoD after treating OTUB1-silenced cells with erastin or DMSO. (**I**) After OTUB1-silenced cells were treated with Lip-1 or DMSO, the protein expression levels of MyoG and MyoD were measured by western blotting. The data represent the means ± SEM (n = 3 independent cell cultures). * *p* < 0.05; ** *p* < 0.01.

Next, we explored the role of OTUB1-mediated ferroptosis in regulating skeletal muscle formation. We assessed the differentiation status of C2C12 myoblasts by measuring the mRNA and protein levels of two myogenic markers, MyoG and MyoD. We discovered that in OTUB1-silenced cells in differentiation medium, the expression levels of MyoG and MyoD were markedly lower (Fig. 3A,B). Next, C2C12 myoblasts were treated with erastin, a potent inducer of ferroptosis, and liproxstatin-1 (Lip-1), an effective inhibitor. We observed that erastin administration exacerbated ferroptosis events in OTUB1-silenced cells, whereas Lip-1 treatment effectively ameliorated this phenomenon (Fig. 3C–F). Interestingly, erastin treatment significantly downregulated the mRNA expression of the myogenic factors Myog and MyoD, whereas Lip-1 treatment markedly upregulated their expression (Fig. 3G). Western blot analysis results also revealed significant decreases in Myog and MyoD protein levels in OTUB1-silenced cells treated with erastin (Fig. 3H); in contrast, Lip-1 treatment markedly increased these protein expression levels (Fig. 3I). These data suggest that OTUB1 can regulate myogenesis through the ferroptosis pathway.

### Relevance of OTUB1 deletion-induced autophagy activation in ferroptosis

**Figure 4:**
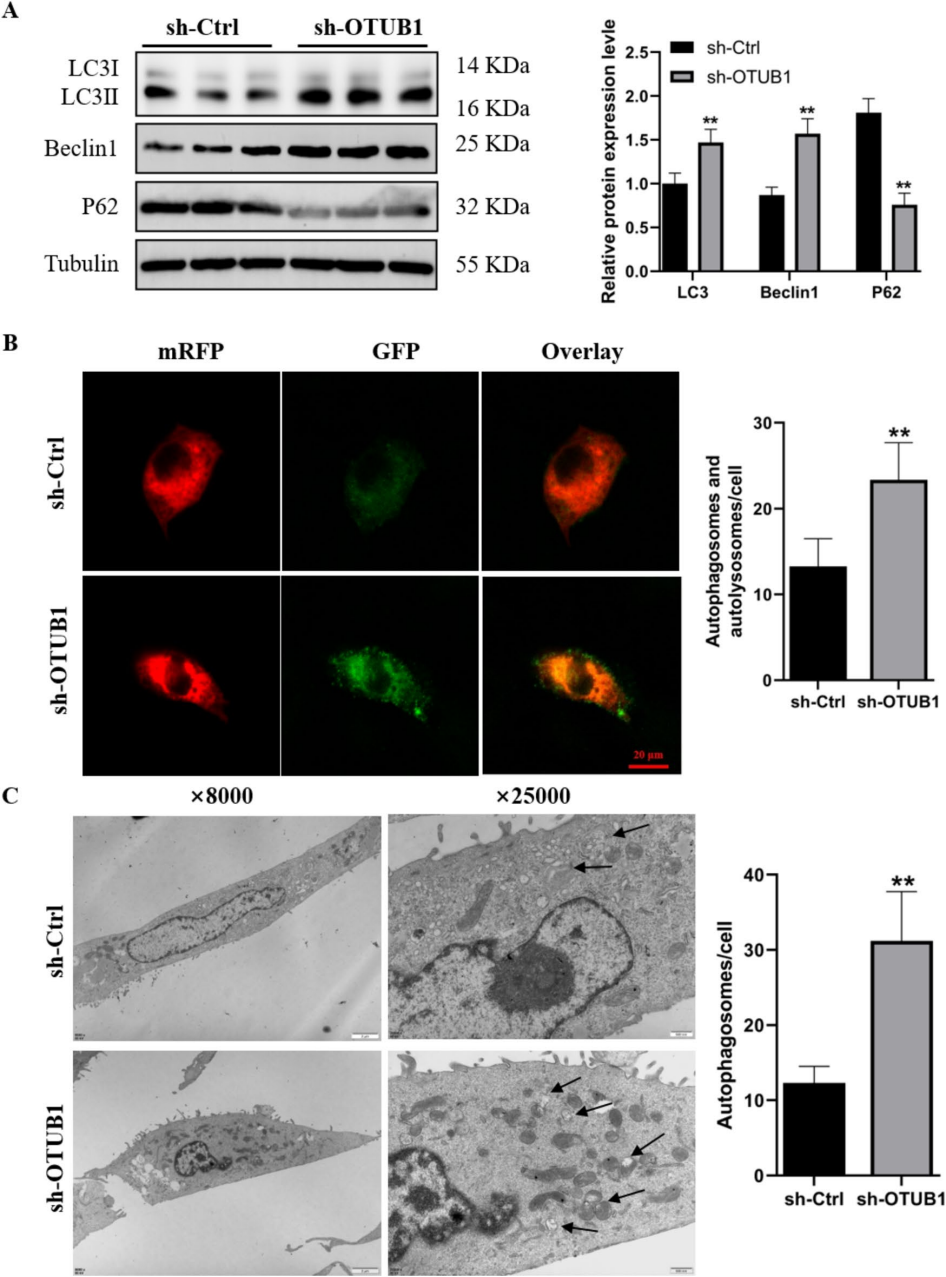
OTUB1 silencing activates autophagy in C2C12 cells. (**A**) The protein expression levels of LC3B, Beclin1, and P62 in OTUB1-silenced and shCtrl cells were measured by western blotting. (**B**) Confocal microscopy was used to detect the number of autophagosomes after mRFP-GFP-LC3 adenovirus transfection in OTUB1-silenced and shCtrl C2C12 cells. Imag-J was employed for quantifying the co-localization of autophagosomes and lysosomes. (C) Using transmission electron microscopy, the ultrastructure of cells transfected with shCtrl or shOTUB1 was examined. The data represent the means ± SEM (n = 3 independent cell cultures). * *p* < 0.05; ** *p* < 0.01.

Ferroptosis is a type of autophagic cell death, as shown by earlier studies. Western blotting research confirmed that OTUB1 silencing increased the LC3-II/LC3-I ratio and Beclin expression, while decreased levels of P62 protein (Fig. 4A). After that, we used the mRFP-GFP-LC3 virus to transfect C2C12 myoblasts and track autophagic flux. We discovered that in OTUB1-silenced cells, the quantity of autophagosomes and autophagolysosomes dramatically increased (Fig. 4B). Finally, OTUB1 shRNA-transfected autophagosome numbers were considerably higher than those of the control group, as demonstrated by transmission electron microscopy (Fig. 4C). These results imply that autophagy activation caused by OTUB1 loss may cause ferroptosis.

### OTUB1 interacts with P62 to modulate autophagy-mediated ferroptosis

**Figure 5:**
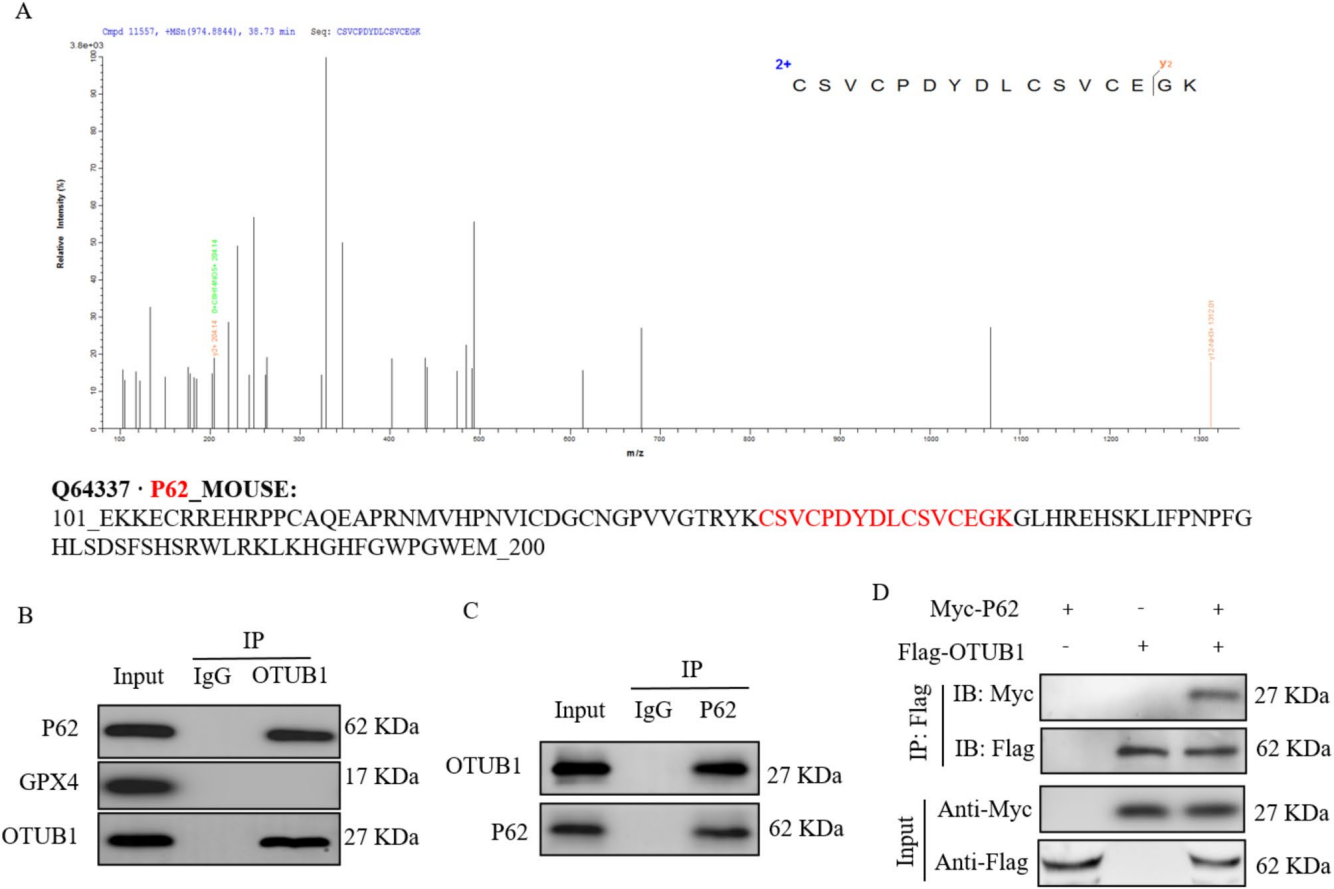
OTUB1 interacts with P62 in C2C12 cells. (**A**) The proteins that bind to OTUB1 were identified using IP/MS analysis; P62 was identified as an interacting protein. (**B**,**C**) Analysis of OTUB1 and P62 using reciprocal co-immunoprecipitation in C2C12 cells. (**D**) Following immunoprecipitation with anti-Flag, the lysates of HEK 293 T cells transfected with Flag-labeled OTUB1 and Myc-labeled P62 were immunoblotted with anti-Myc (P62) and anti-Flag (OTUB1). Following immunoprecipitation with an anti-Flag antibody, lysates of HEK 293T cells transfected with Flag-tagged OTUB1 and Myc-tagged P62 were immunoblotted with anti-Myc (P62) and anti-Flag (OTUB1).

We investigated the protein interactome of OTUB1 using immunoprecipitation coupled with mass spectrometry (IP/MS) in order to clarify the underlying mechanism by which OTUB1 knockdown triggers autophagy during ferroptosis. P62 was recognized by the IP/MS analysis as a potential OTUB1 interacting protein (Fig. 5A). Next, we conducted a Co-IP analysis to validate the IP/MS findings. OTUB1 was found to precipitate P62 in C2C12 cells, while GPX4 (another protein known for its interaction with OTUB1 and ferroptosis regulation) and control IgG did not exhibit any precipitation (Fig. 5B). It was verified by reverse Co-IP that P62 strongly precipitated OTUB1 in C2C12 cells (Fig. 5C). Using 293 T cells and proteins that had been epitope-tagged, a second Co-IP test was carried out. In line with our prior findings, the 293T cells demonstrated effective co-precipitation of Myc-labeled P62 and Flag-labeled OTUB1 (Fig. 5D). These results imply that autophagy-mediated ferroptosis is regulated by the connection between OTUB1 and P62.

### OTUB1 inhibits the ubiquitination and degradation of P62

**Figure 6:**
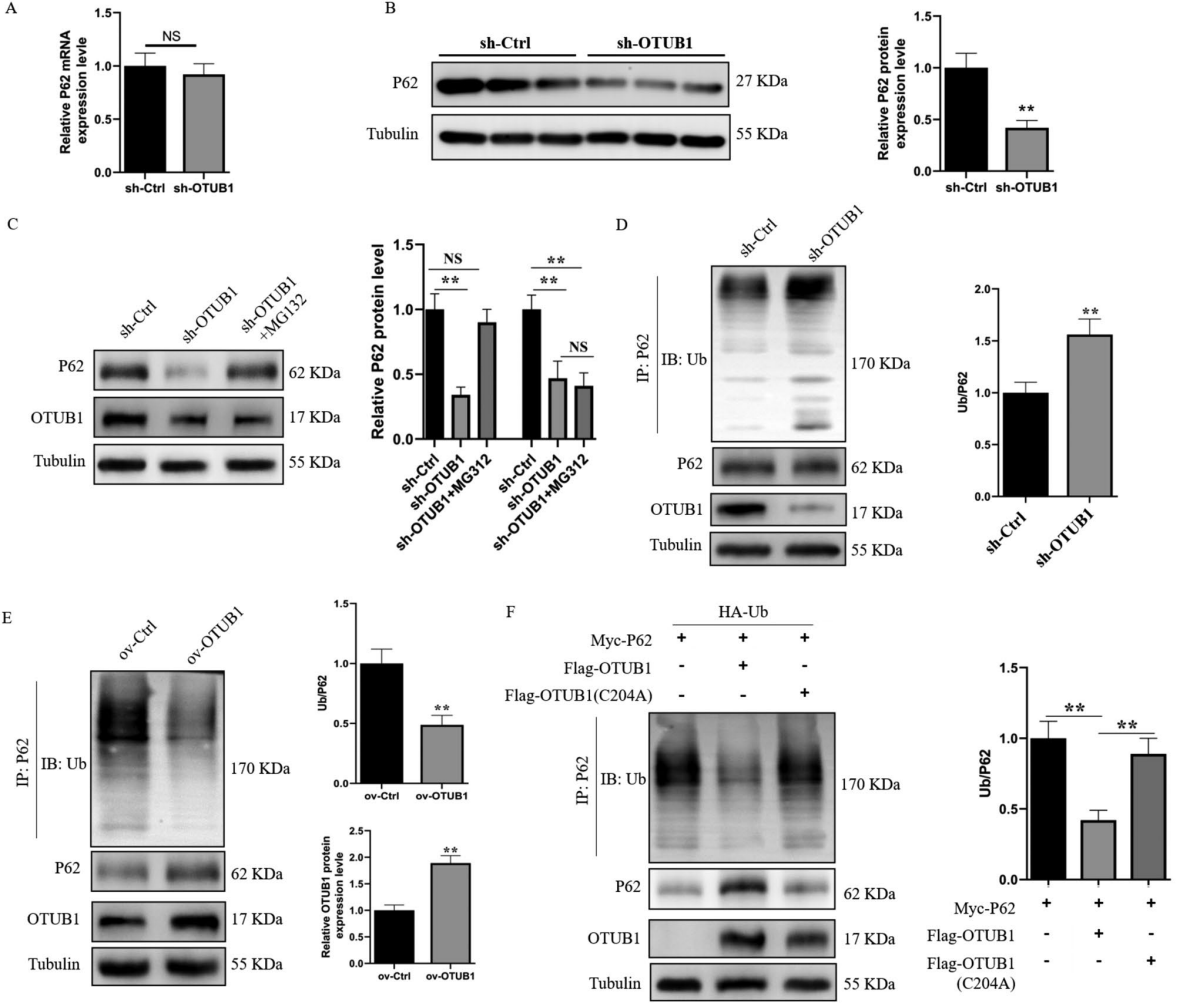
OTUB1 inhibits P62 ubiquitination and degradation. (**A**,**B**) OTUB1-silenced and shCtrl cells' P62 mRNA and protein levels were examined using qRT-PCR and western blot techniques. (**C**) Western blot analysis of OTUB1 and P62 in shCtrl and OTUB1-silenced cells treated or not with the proteasome inhibitor MG132. (**D**) The specified shRNA and HA-Ub were co-transfected into C2C12 cells treated with MG132. The cell lysates were then subjected to immunoprecipitation (IP) using a P62 antibody and subsequently immunoblotted (IB) with HA-Ub and P62 antibodies, all in the presence of MG132. (**E**) C2C12 cells were co-transfected with the OTUB1 overexpression or control plasmid and HA-Ub, and cell lysates were subjected to immunoprecipitation (IP) using a P62 antibody, followed by immunoblotting (IB) with HA-Ub and OTUB1 antibodies. (**F**) Following immunoprecipitation and anti-Myc immunoblotting, the lysates of C2C12 cells transfected with HA-tagged Ub and Myc-tagged P62, as well as Flag-tagged OTUB1 (WT) or Flag-tagged OTUB1 (C204A), were probed with anti-HA and anti-Myc antibodies. The data represent the means ± SEM (n = 3 independent cell cultures). * *p* < 0.05; ** *p* < 0.01.

Next, we looked into how P62 expression was affected by OTUB1 knockdown in C2C12 cells. Compared to the control conditions, OTUB1 knockdown significantly reduced P62 protein levels, while no discernible effects on P62 mRNA levels were observed (Fig. 6A,B). This finding suggests that the regulation of P62 by OTUB1 occurs post-transcriptionally, potentially influencing P62 protein stability. The addition of the proteasome inhibitor MG132 reversed the decline in P62 protein level after OTUB1 was depleted (Fig. 6C). This suggests that OTUB1 may regulate P62 protein stability through the ubiquitin proteasome system. Subsequently, we sought to examine the potential regulatory role of OTUB1 in the ubiquitination and degradation of P62. Comparative analyses revealed that knockdown of OTUB1 significantly enhanced P62 ubiquitination and reduced P62 protein levels (Fig. 6D). Moreover, our findings demonstrate that OTUB1 overexpression significantly inhibited P62 ubiquitination, while increasing P62 protein levels (Fig. 6E). Then, the cysteine residue at position 204 of OTUB1 was substituted with alanine to generate a catalytically inactive variant of OTUB1 protein. In order to verify the impact of OTUB1 on P62 ubiquitination, we co-transfected Flag-OTUB1 (wild-type or C204A mutant), Myc-P62, and HA-Ub into HEK 293T cells. The ubiquitination of P62 was efficiently prevented by overexpressing wild-type OTUB1, an effect that was counteracted by the C204A mutant of OTUB1 (Fig. 6F). These findings suggest that OTUB1 controls P62 stability in C2C12 cells, which affects the development of skeletal muscle.

### OTUB1 affects ferroptosis pathway by mediating P62 to regulate myogenesis

**Figure 7:**
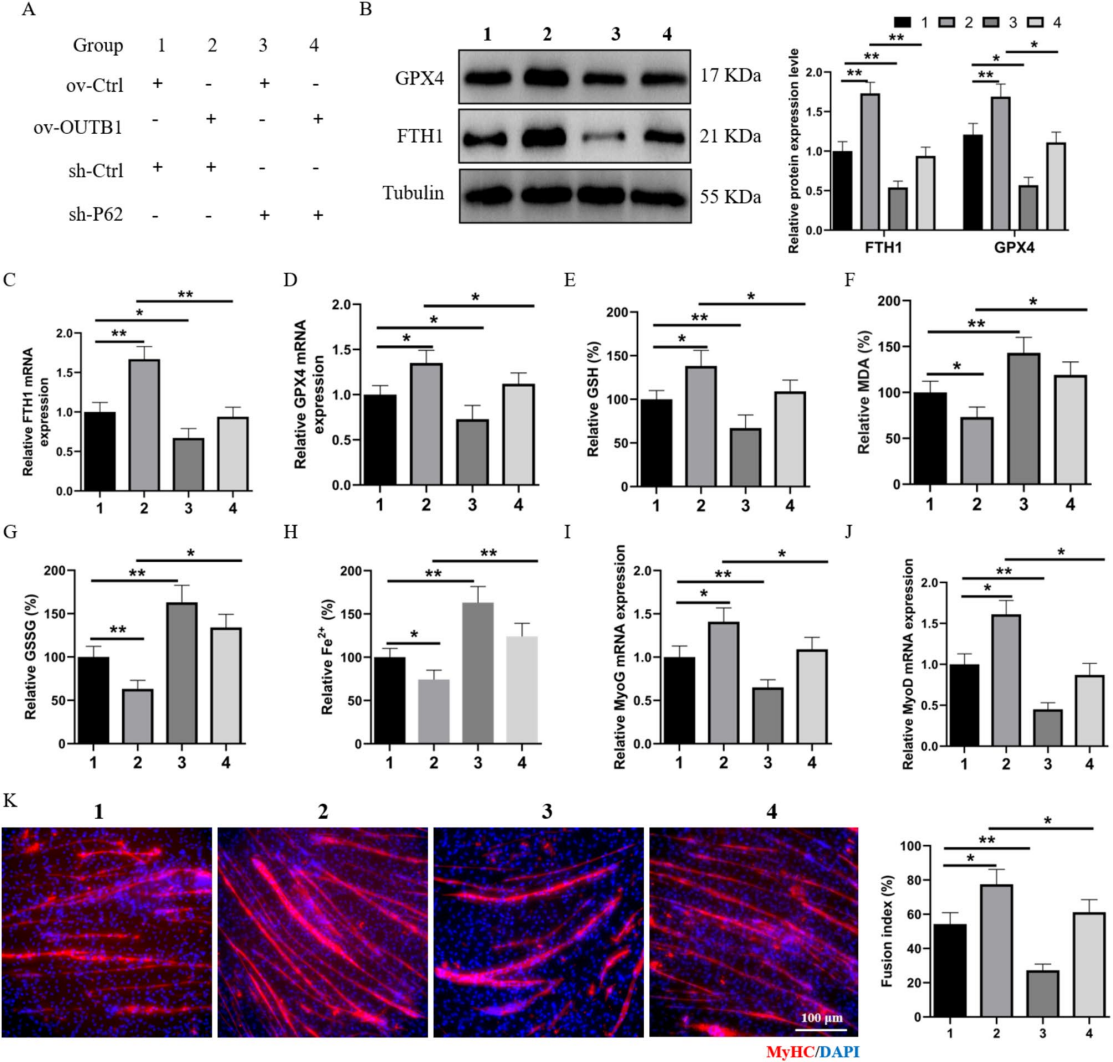
OTUB1 mediates P62 to inhibit ferroptosis and rescue myogenesis. (**A**) Comprehensive details of the experimental groups in this phase of the study. (**B**) Western blot demonstrating the ferroptosis-associated proteins' FTH1 and GPX4 expression in the designated treated cells. (**C**,**D**) qRT-PCR analysis of GPX4 and FTH1 mRNA expression in the indicated treated cells. (**E**–**H**) GSH, MDA, GSSG, and Fe^2+^ concentrations were measured in the indicated treated cells. (**I**,**J**) MyoG and MyoD mRNA expression in the appropriate treated cells was examined using qRT-PCR. (**K**) Histochemical staining of the indicated treated cells using immunofluorescence. The data represent the means ± SEM (n = 3 independent cell cultures). *p < 0.05; **p < 0.01.

We then investigated whether OTUB1 could selectively target P62 to inhibit ferroptosis and promote skeletal muscle development. We co-transfected an OTUB1 overexpression vector and a P62-targeting shRNA vector. The experimental group details are presented in Fig. 7A. We observed that the absence or presence of ov-OTUB1 in C2C12 cells transfected with P62 resulted in the activation of ferroptosis events (Fig. 7B–H). Furthermore, we observed that P62 silencing significantly downregulated the myogenic genes MyoG and MyoD, in both the presence and absence of OTUB1 overexpression (Fig. 7I,J). Immunofluorescence analysis of MyHC revealed that OTUB1 enhanced myoblast differentiation into myotubes, but the myotube differentiation rate was attenuated upon P62 silencing (Fig. 7K). These data suggest that p62 silencing inhibits myogenesis and promotes ferroptosis in ov-OTUB1 cells.

## Discussion

In the present study, we have show that skeletal muscle diseases are associated with ferroptosis. Ferroptosis activation can suppress the expression of the myogenic genes MyoG and MyoD, thereby impeding the differentiation of myoblasts into myotubes. Furthermore, we discovered OTUB1 to be a new ferroptosis regulator. Mechanistically, OTUB1 exerts its inhibitory effect on autophagy by promoting P62 stabilization and suppressing the autophagic degradation of ferritin; these effects ultimately inhibit iron-dependent ferroptotic cell death. Therefore, we suggest that OTUB1 may be a useful therapeutic target for the management or prevention of disorders involving the skeletal muscle.

Mounting evidence substantiates the pivotal role of OTUB1 in the regulation of lung development, DNA damage repair, respiratory control, and cancer metastasis^28–30^. Since OTUB1 targets crucial ferroptosis regulatory components, numerous studies have indicated that it plays a significant regulatory function in the ferroptosis pathway. Li, et al. reported that OTUB1 plays a crucial role in regulating GPX4 protein stability, which inhibits ferroptosis and promotes metastasis in gastric cancer^31^. Liu, et al. found that OTUB1 plays a pivotal role in regulating SLC7A11 stability and modulating the CD44-mediated effect on ferroptosis in human malignancies^32^. Additionally, Huang, et al. discovered that the m6A demethylase FTO confers resistance to nasopharyngeal carcinoma by promoting OTUb1-mediated anti-ferroptosis^33^. These studies all confirm that OTUB1 is a negative regulator of ferroptosis that can promote cell viability and accelerate tumor growth. In this study, we observed that OTUB1 expression was markedly inhibited upon treatment with the ferroptosis inducer RSL3. Furthermore, OTUB1 knockdown enhanced lipid peroxidation and iron content accumulation, thereby activating C2C12 myoblast ferroptosis and ultimately impeding myogenesis. This study is also the first to identify the important role of OTUB1 in skeletal muscle. Additionally, our data suggest that skeletal muscle development induced by OTUB1 through the ferroptosis pathway may do duty for a potential target for treating skeletal muscle-related diseases.

Interestingly, numerous studies have demonstrated a significant correlation between ferroptosis and autophagy^34^. It is thought that autophagy is an upstream mechanism that causes ferroptosis by controlling the production of reactive oxygen species and cellular iron homeostasis^35^. We observed that OTUB1 silencing enhances autophagosome production and augments autophagy flux, thereby revealing a potential mechanistic link between OTUB1 and ferroptosis promotion. To further elucidate the underlying mechanism by which OTUB1 silencing activates autophagy during ferroptosis, we conducted IP/MS analyses and identified a direct interaction between the OTUB1 and P62 proteins in C2C12 cells. This finding implies that P62 might also govern the growth of skeletal muscle by limiting ferroptosis via autophagy.

P62 is essential to the autophagy system because it directly interacts with Atg8/LC3 to promote autophagy-mediated destruction of ubiquitinated protein aggregates^36^. Numerous studies conducted recently have revealed the critical function of P62 in the complex regulation of ferroptosis^37^. By inactivating Kelch-like ECH-associated protein 1 (Keap1), P62 expression inhibits NRF2 degradation and promotes its subsequent nuclear accumulation, which obstructs the ferroptosis process^38^. Zhao, et al. discovered that inhibiting autophagy upregulated P62 expression, which subsequently interacted with Keap1 to facilitate Nrf2 nuclear translocation; this translocation upregulated the downstream target ferroptosis proteins, including ferritin heavy chain (FTH), ferritin light chain (FPN), and heme oxygenase-1 (HO-1)^39^. These studies suggest that P62 can inhibit ferroptosis events by regulating ferritin expression through the Keap1-Nrf2 signaling axis. In this study, we demonstrated a stable interaction between OTUB1 and P62 that eliminates P62 ubiquitination and subsequently reduces P62 degradation in C2C12 cells. Increased P62 protein levels inhibit autophagy-mediated ferroptosis, thereby promoting skeletal muscle development.

## Conclusions

**Figure 8:**
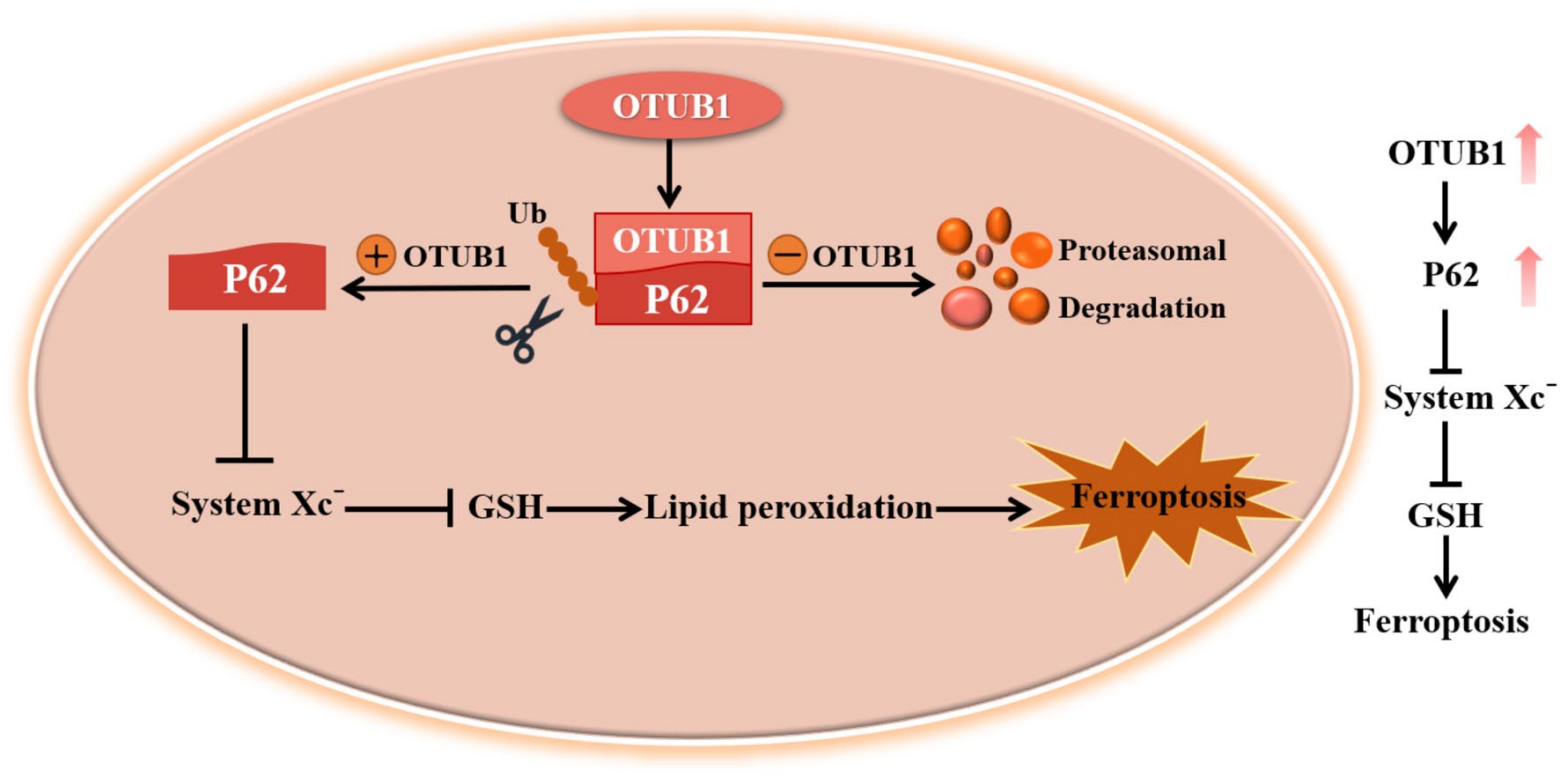
OTUB1 modulates skeletal muscle development by regulating ferroptosis through deubiquitinating P62. Upregulation of OTUB1 inhibits the System Xc- by stabilizing P62, thereby inhibiting ferroptosis induced by lipid peroxidation and ultimately protecting skeletal muscle development.

In summary, our findings indicate a causative role of OTUB1 in ferroptosis inhibition, suggesting that the absence of OTUB1 activates autophagy-dependent ferroptosis in skeletal muscle that induces significant muscle loss and weakness. We thus propose that OTUB1 may play a dual role in both cytoarchitecture and signal transduction during ferroptosis (Fig. 8). Future research is required to fully understand the precise mechanisms and degree to which OTUB1-deficient myopathies are caused by ferroptosis dysregulation; investigating autophagy-mediated ferroptosis in muscle cells obtained from animal models will be essential to achieving this goal.

### Supplementary Information

Supplementary Information.

## Data Availability

The original contributions presented in the study are included in the article, further inquiries can be directed to the corresponding authors.
