# Supplementary material for: OTUB1 regulates ferroptosis to inhibit myoblast differentiation into myotubes by deubiquitinating P62
Source: Sci Rep. 2024 Jul 8;14:15696. doi: 10.1038/s41598-024-66868-3 (PMC11231240; doi:10.1038/s41598-024-66868-3)

Figure 1J

OTUB1 MyoG MyoD Tubulin


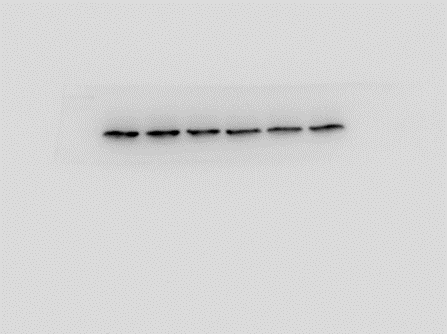

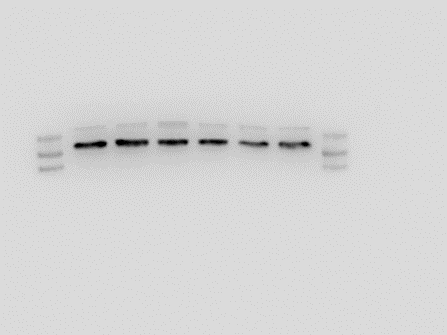

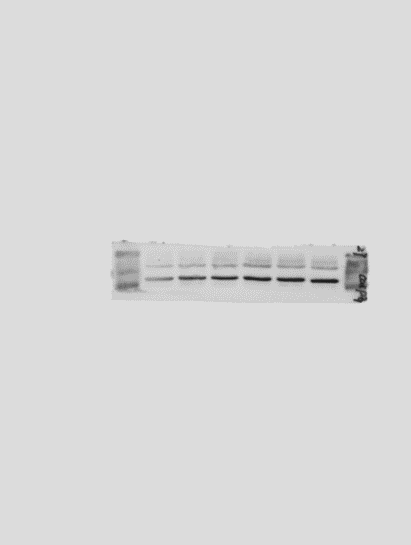

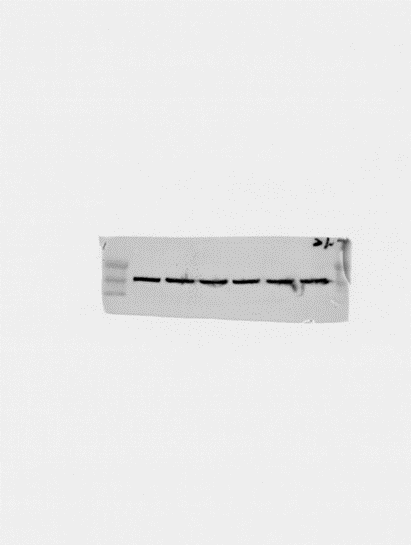


Figure 2B

OUTB1 Tubulin







Figure 2J

GPX4 FTH1 Tubulin


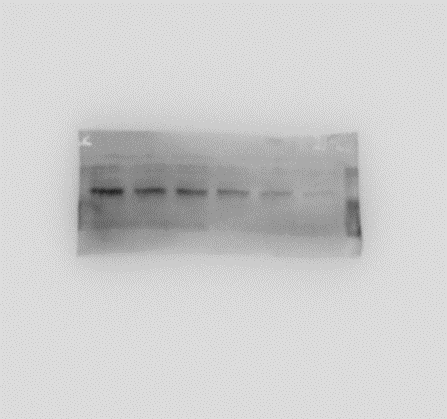

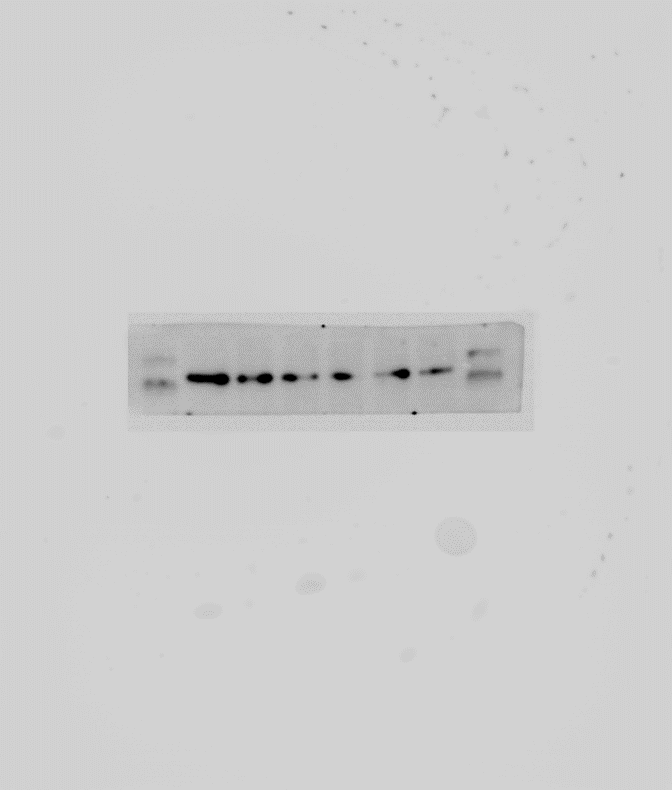

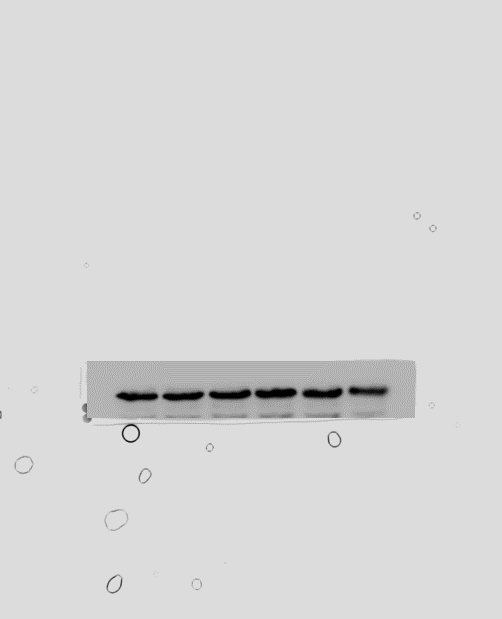


Figure 3B

MyoG MyoD Tubulin









Figure 3H

MyoG MyoD Tubulin




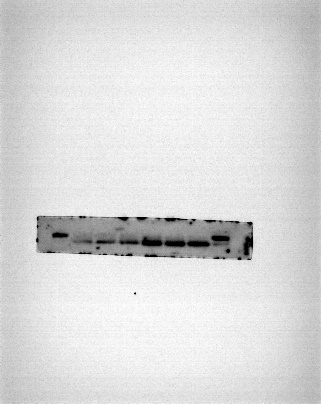

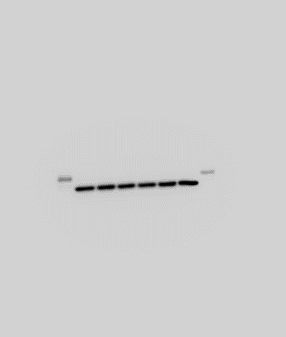


Figure 3I

MyoG MyoD Tubulin









Figure 4A

LC3-Ⅱ/LC3-I Beclin1 P62 Tubulin











Figure 5B

P62 GPX4 OTUB1









Figure 5C

OTUB1 P62







Figure 5D

IB: Myc IB: Flag Anti-Myc Anti-Flag








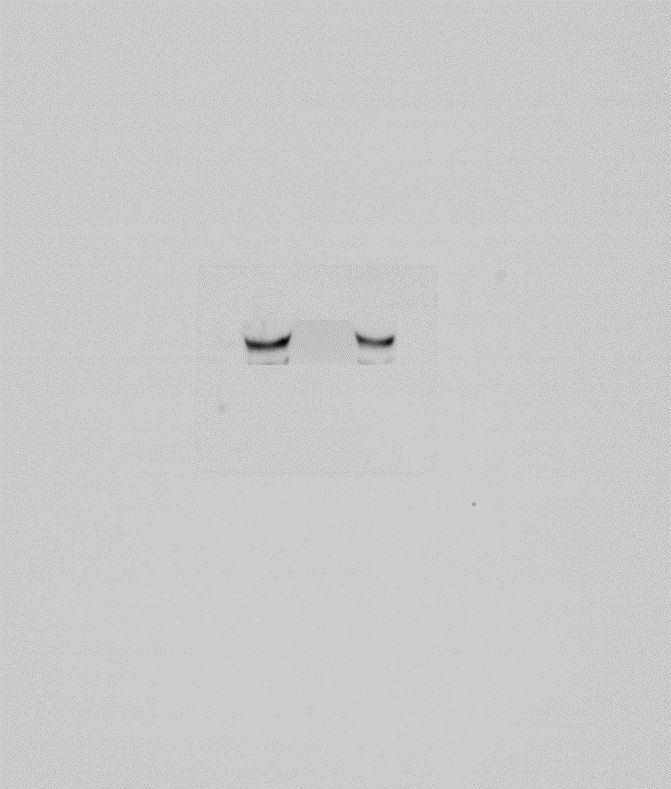


Figure 6B

P62 Tubulin


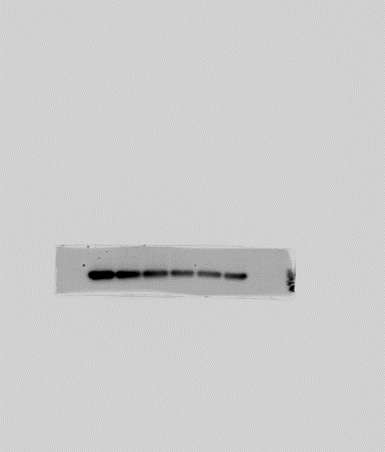

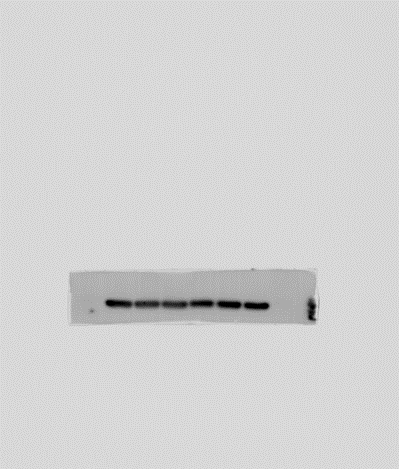


Figure 6C

P62 OTUB1 Tubulin


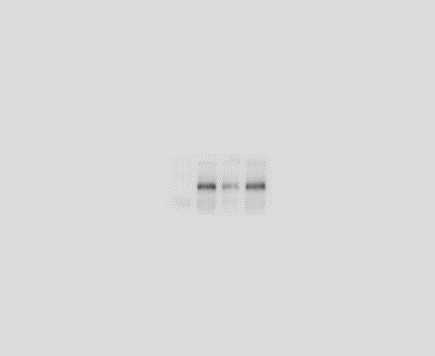

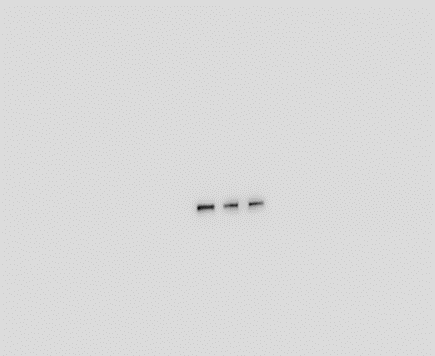

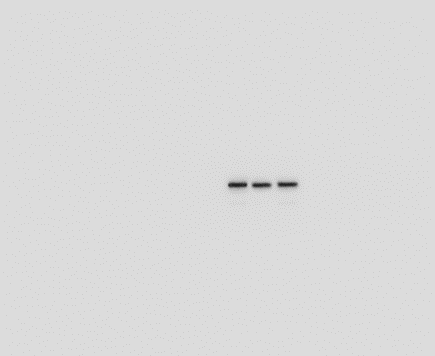


Figure 6D

IB: Ub P62 OTUB1 Tubulin


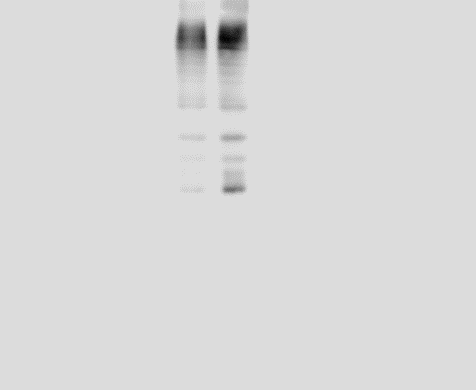

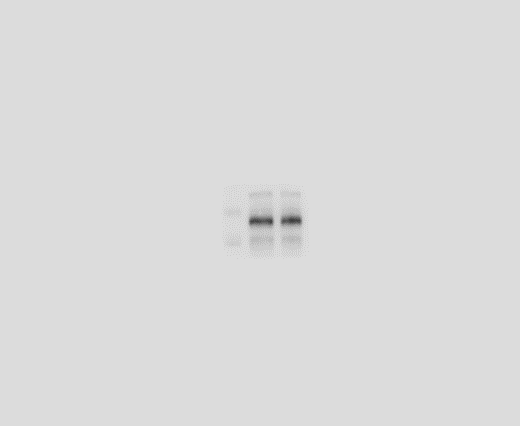

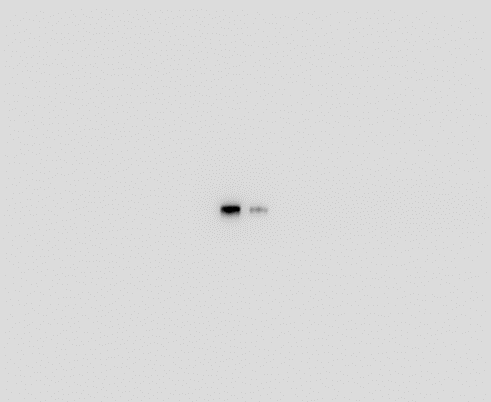

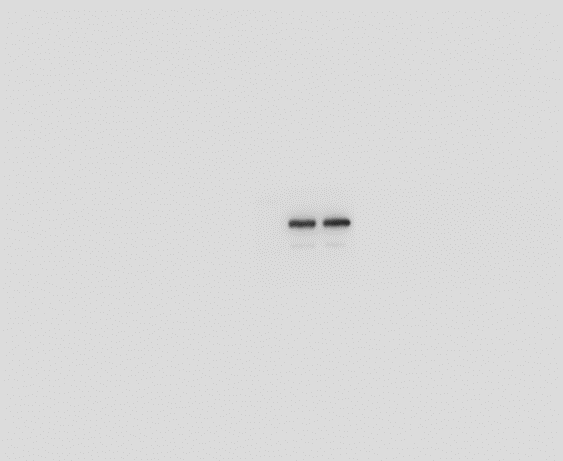


Figure 6E

IB: Ub P62 OTUB1 Tubulin


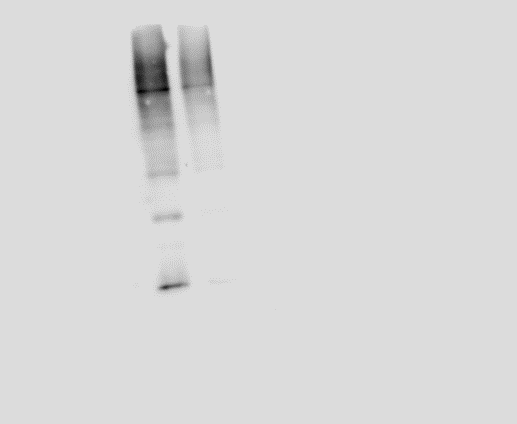

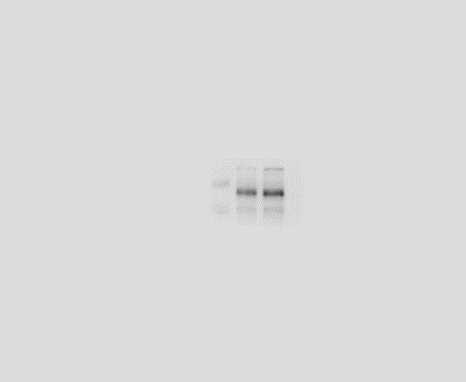

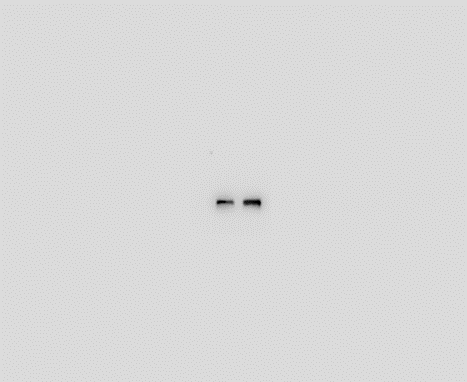

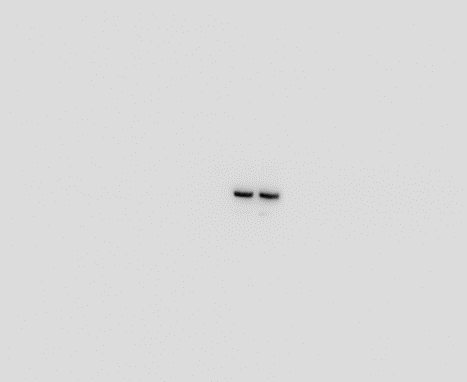


Figure 6F

IB: Ub P62 OTUB1 Tubulin


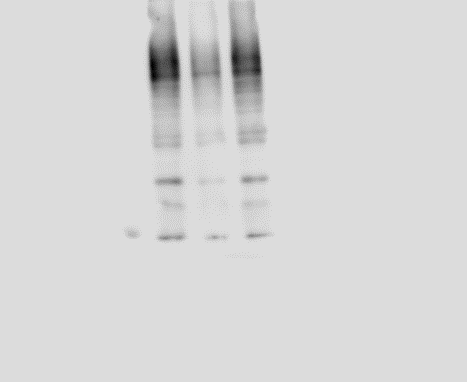

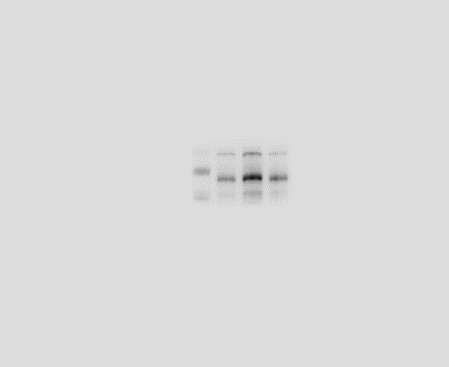

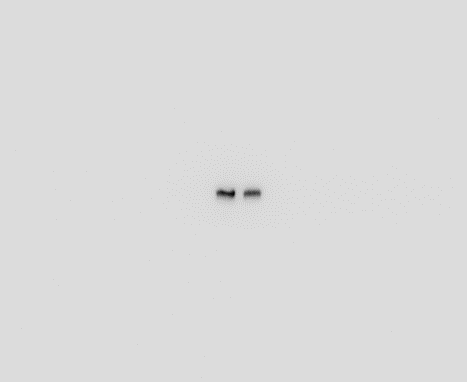

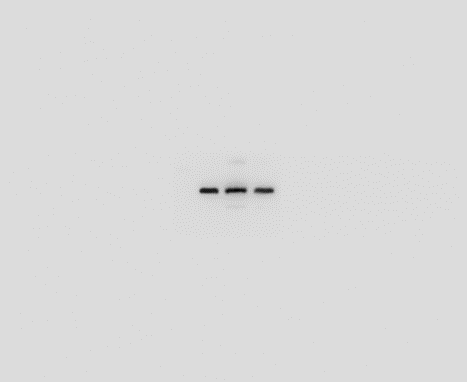


Figure 7B

GPX4 FTH1 Tubulin


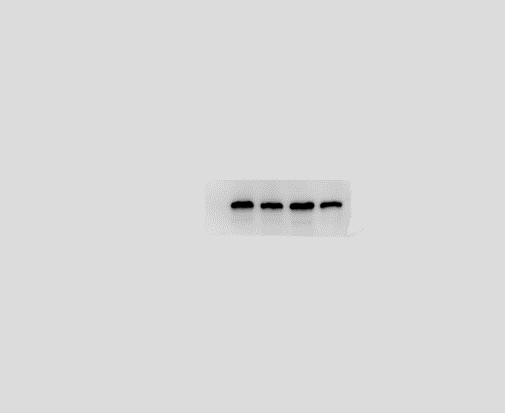

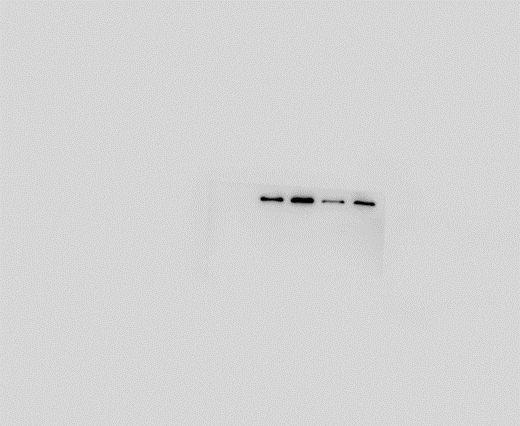

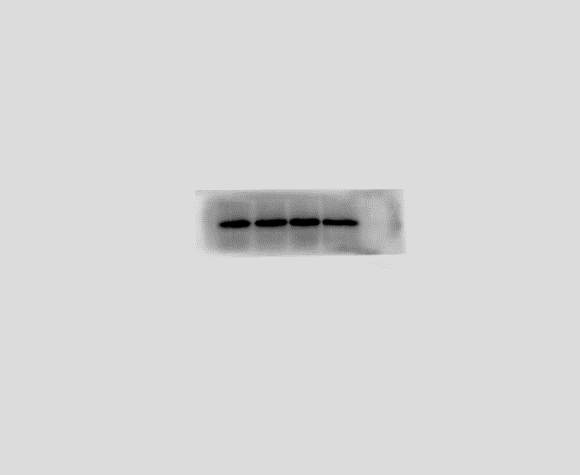

Supplement: Supplementary file 1 — Supplementary Information. [file 41598_2024_66868_MOESM1_ESM.docx]
